# Cybersecurity of Smart Sensing Insole Systems: A Threat Modelling Approach to Understanding Security Vulnerabilities

**DOI:** 10.3390/s26196298

**Published:** 2026-10-05

**Authors:** Padideh Choobdar, Nigel Davies, Emma D. Wilson, Steve Hodges, Edward B. Jude, Matthew Bradbury, Neil D. Reeves

**Affiliations:** 1 Medical School, Faculty of Health and Medicine, Lancaster University, Lancaster LA1 4AT, UK; p.choobdar@lancaster.ac.uk; 2 School of Computing and Communications, Faculty of Science and Technology, Lancaster University, Lancaster LA1 4WA, UK; n.a.davies@lancaster.ac.uk (N.D.); e.d.wilson1@lancaster.ac.uk (E.D.W.); steve.hodges@lancaster.ac.uk (S.H.); m.s.bradbury@lancaster.ac.uk (M.B.); 3 Tameside and Glossop Integrated Care NHS Foundation Trust, Ashton-Under-Lyne, Manchester OL6 9RW, UK; edward.jude@tgh.nhs.uk

**Keywords:** threat modelling, wearable medical device, STRIDE, IoT, smart insole, diabetes, hacking

## Abstract

Smart sensing insoles are emerging medical IoT devices used primarily in diabetes care to monitor foot pressure, temperature and gait for preventing diabetic foot ulcers and lower-limb amputation. Despite growing healthcare use, the cybersecurity threats to these systems have received limited systematic analysis in the literature. This study aimed to provide a detailed threat modelling analysis of smart insole systems. The study developed a representative smart insole system architecture using public information, the literature, and manufacturer input. A data flow diagram mapped devices, processes, data stores, data flows, and trust boundaries across sensor, edge/mobile, cloud and clinician zones. Potential cybersecurity threats were analysed using STRIDE, MITRE ATT&CK mapping and attack trees. The modelled analysis identified potential threats across all STRIDE categories: spoofing, tampering, repudiation, information disclosure, denial of service and elevation of privilege. Three attacker goals were modelled: stealing health data, reducing trust in the technology and inflicting patient harm. Key modelled threats include BLE interception, weak authentication, data tampering, service disruption, log deletion and manipulation of clinical outputs. Commercial systems varied substantially in their security features. The modelled findings show that cybersecurity threats to smart insoles extend beyond data privacy to include threats to patient safety. Disrupted alerts, altered pressure data or unavailable monitoring could delay clinical action and increase diabetic foot ulcer and amputation risk. The study highlights the need for secure-by-design systems, including BLE encryption, strong authentication, audit logging and clearer regulatory/procurement standards.

## 1. Introduction

Digital wearable healthcare technologies are playing an increasingly vital role in helping people manage and prevent a wide range of diseases [1,2,3]. As healthcare systems face the challenges of growing populations, limited staffing, and financial pressures, these technologies are becoming increasingly adopted for delivering more efficient and accessible healthcare. This is particularly the case in the UK, where the government’s 10-year health plan seeks to shift healthcare from hospitals to communities/homes, from analogue to digital, and from sickness to prevention [4]. All three of these ‘shifts’ in the UK government’s health plan are underpinned by digital healthcare technologies, underlining the need for not only effective but also secure technologies [5].

Diabetes is a disease of rising global prevalence and an example where digital healthcare technologies are being increasingly leveraged to help with better disease management and to reduce further complications [1,2,6]. For example, continuous glucose monitors and insulin pumps are used to help people with diabetes maintain appropriate blood glucose levels and reduce the risk of complications arising from diabetes [7,8]. Sensing insoles/socks are gaining traction in healthcare systems as an effective tool for preventing lower limb complications, including diabetic foot ulcers, which often result in lower limb amputation. These insole systems have demonstrated clinical efficacy for foot ulcer prevention in high-risk diabetes patients, avoiding lower limb amputations and highlighting their potential to transform healthcare [6,9]. In the UK and Europe, smart sensing insoles are typically categorised as Class I medical devices and can be considered Internet of Things (IoT) medical systems due to their local connectivity with hubs and mobile devices such as smartphones, and through their connection to the internet and cloud servers [6,9,10,11]. As their use and integration within global healthcare systems expands, understanding cybersecurity vulnerabilities and ensuring robust cybersecurity mitigations is paramount.

Addressing cybersecurity vulnerabilities and identifying effective mitigation strategies requires a comprehensive understanding of system architecture, data flow, potential threat actors, and attack vectors [12]. Although there have been several studies addressing these aspects in continuous glucose monitors and insulin pumps [13,14,15,16], these critical aspects remain unexplored for smart sensing insole systems. The focus on these medical IoT systems is justified by their proliferation across global health services, despite no published comprehensive system-level cybersecurity threat model specifically focused on smart sensing insoles, therefore representing a key gap in scientific understanding.

Unlike traditional computers and network systems with robust operating systems, smart sensing insoles are resource-constrained digital devices with limited processing power and energy capacity, presenting unique cybersecurity challenges [12]. Their connection to cloud servers and other systems increases the attack surface, potentially exposing broader healthcare infrastructure to cyber threats. These insoles wirelessly connect to mobile devices via Bluetooth or other wireless protocols. Over-the-air (OTA) wireless protocols have previously demonstrated cybersecurity vulnerabilities in other digital healthcare devices, including continuous glucose monitors [16], insulin delivery systems [13,14] and heart rhythm/ECG monitors [13,17]. Specifically, these vulnerabilities often arise from unencrypted data transmission, insecure communication protocols, and insufficient authentication measures, all of which expose resource-limited digital IoT medical technologies to cyber risk [14]. Cybersecurity threats in wireless communications can lead to privacy breaches, where sensitive medical data is intercepted; denial-of-service attacks, which compromise system availability for legitimate users; and integrity attacks, where intercepted data is maliciously manipulated to disrupt device function. Such attacks pose serious risks to patient safety and underscore the critical need for rigorous cybersecurity measures in IoT medical technologies, including the unexplored area of smart sensing insole systems.

Threat modelling is a systematic approach for analysing and identifying cybersecurity threats to a system, network or application. It requires a detailed understanding of system architectures, devices/assets and data flows within the system to enable mapping of potential attack vectors and an understanding of cybersecurity vulnerabilities. As well as understanding potential vulnerabilities, this process enables the development of potential security mitigations. Although threat modelling might be more commonly applied to large-scale networks, it has previously been applied to IoT health devices [18].

The STRIDE framework is a structured threat modelling methodology that employs a systematic approach to identifying and analysing cybersecurity threats across a range of system components. By decomposing system architecture, including sensors, gateways, IoT devices, cloud infrastructure and mobile devices, it enables a thorough understanding of security threats specific to each component. Threats are classified into six distinct categories: spoofing, tampering, repudiation, information disclosure, denial of service, and elevation of privilege [19,20]. Whilst STRIDE provides an effective framework for threat classification, it does not define the overall threat modelling process. Consequently, in this study, STRIDE is employed predominantly as a classification layer, whilst the broader threat model is developed through system-driven analysis encompassing adversary identification, attack tree construction, and attack-path mapping. Among the established models, the STRIDE framework is widely used to categorise and address cybersecurity threats systematically and can be considered the most appropriate for application to digital healthcare technology [18].

**Figure 1:**
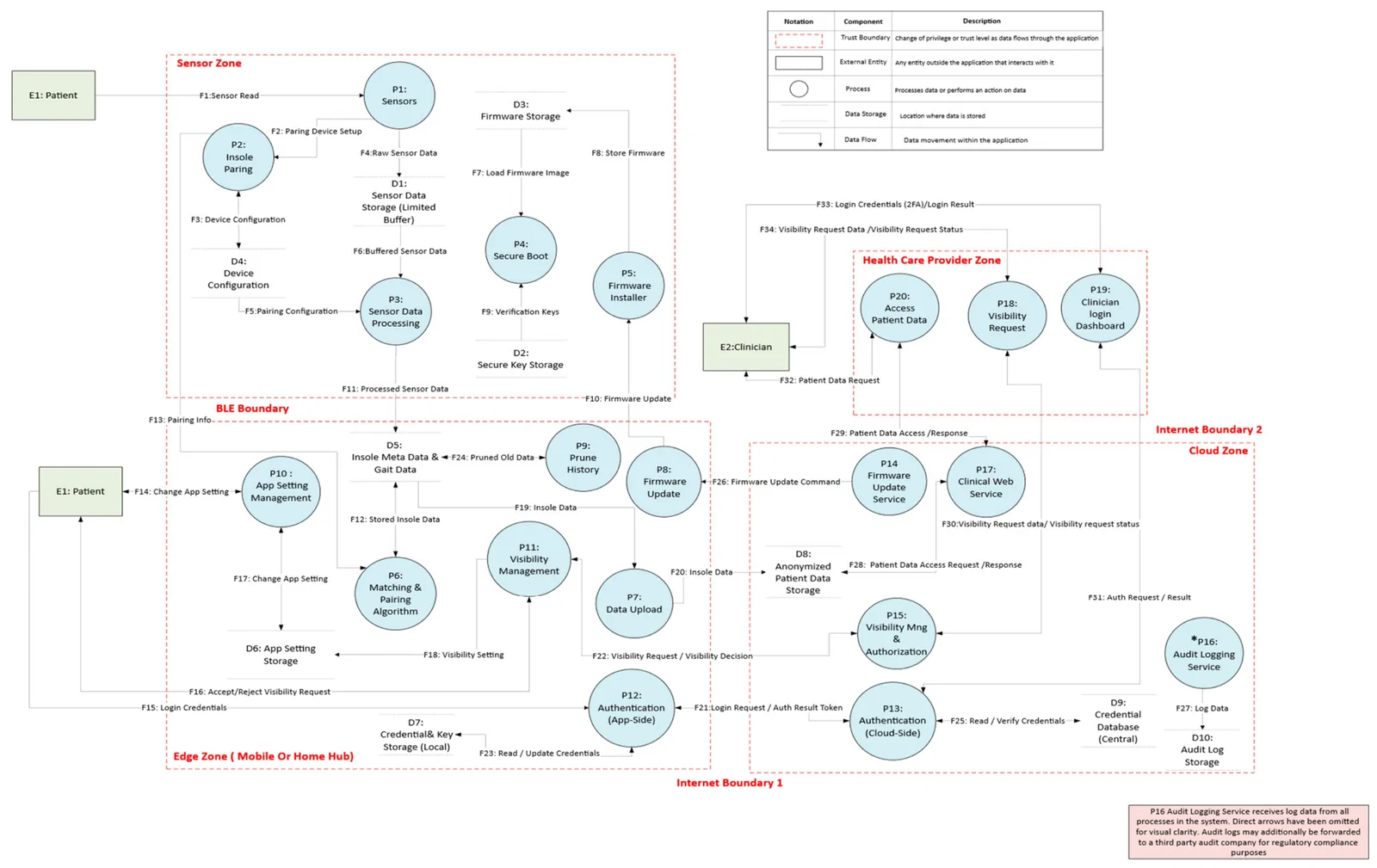
Data flow diagram (DFD) showing the medical IoT smart sensing insole system architecture. The system architecture is divided into four trust zones: (1) the Sensor Zone (sensing insole/sock device), (2) Edge Zone (smartphone or home hub), (3) Cloud Zone and (4) Healthcare Provider Zone. Data moves within and across these zones via two communication boundaries: the BLE Boundary (between the Sensor and Edge zones) and the Internet Boundary (between the Edge and Cloud zones, and between the Cloud and Healthcare Provider zones). The diagram shows processes (P), data storage (D), data flows (F) and external entities (E; the patient/user and the clinician).

**Figure 2:**
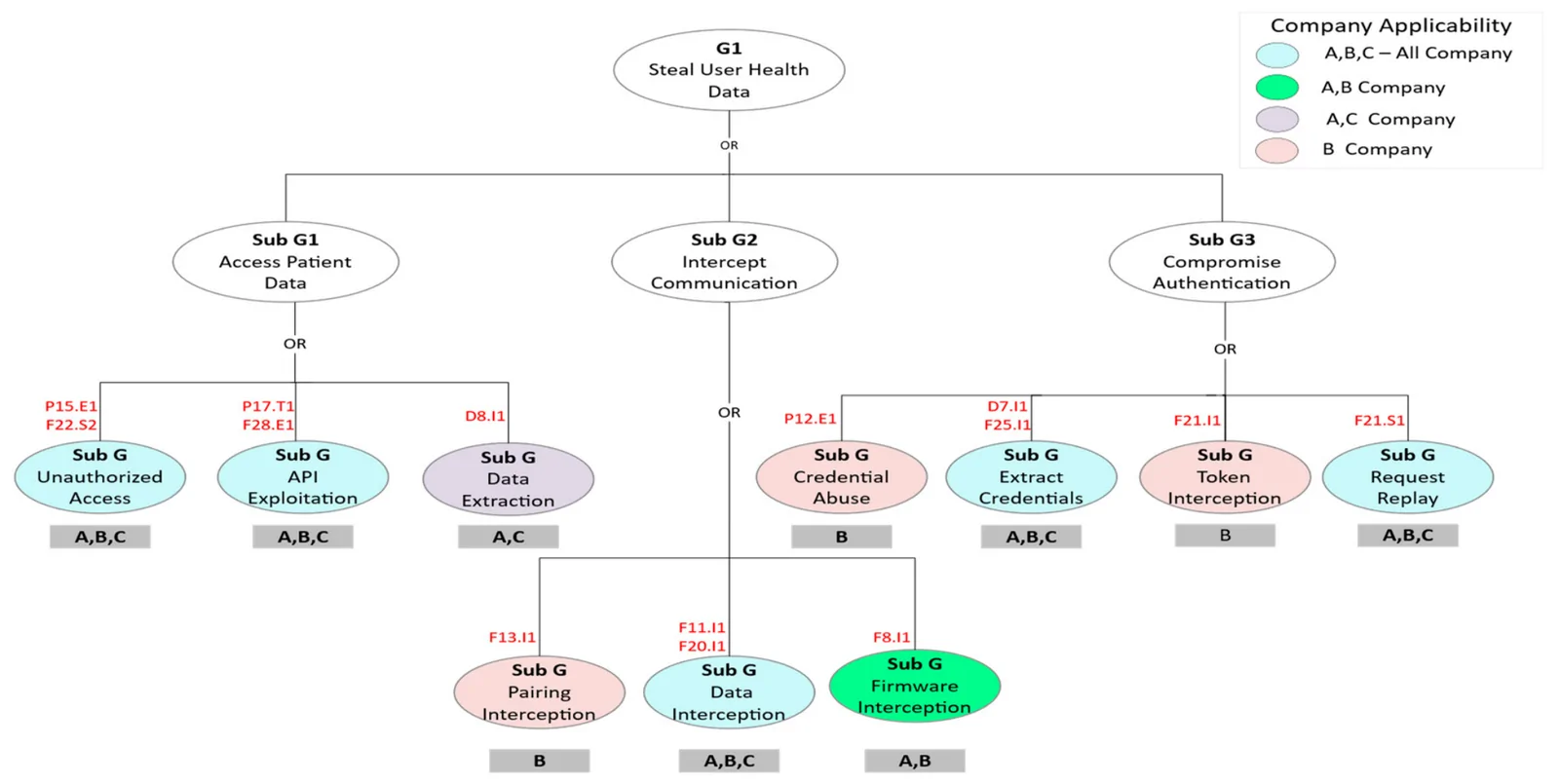
Attack tree for the sensing insole system representing attacker Goal 1: stealing user health data. The tree illustrates how an adversary may directly access patient data (Sub G1), intercept data in transit (Sub G2), or compromise authentication mechanisms (Sub G3) to obtain sensitive gait and clinical information without authorisation. Leaf node identifiers follow the notation defined in Section 4.1; for example, P15.E1 denotes the first elevation of privilege threat identified for process 15 (visibility management) in the STRIDE analysis.

**Figure 3:**
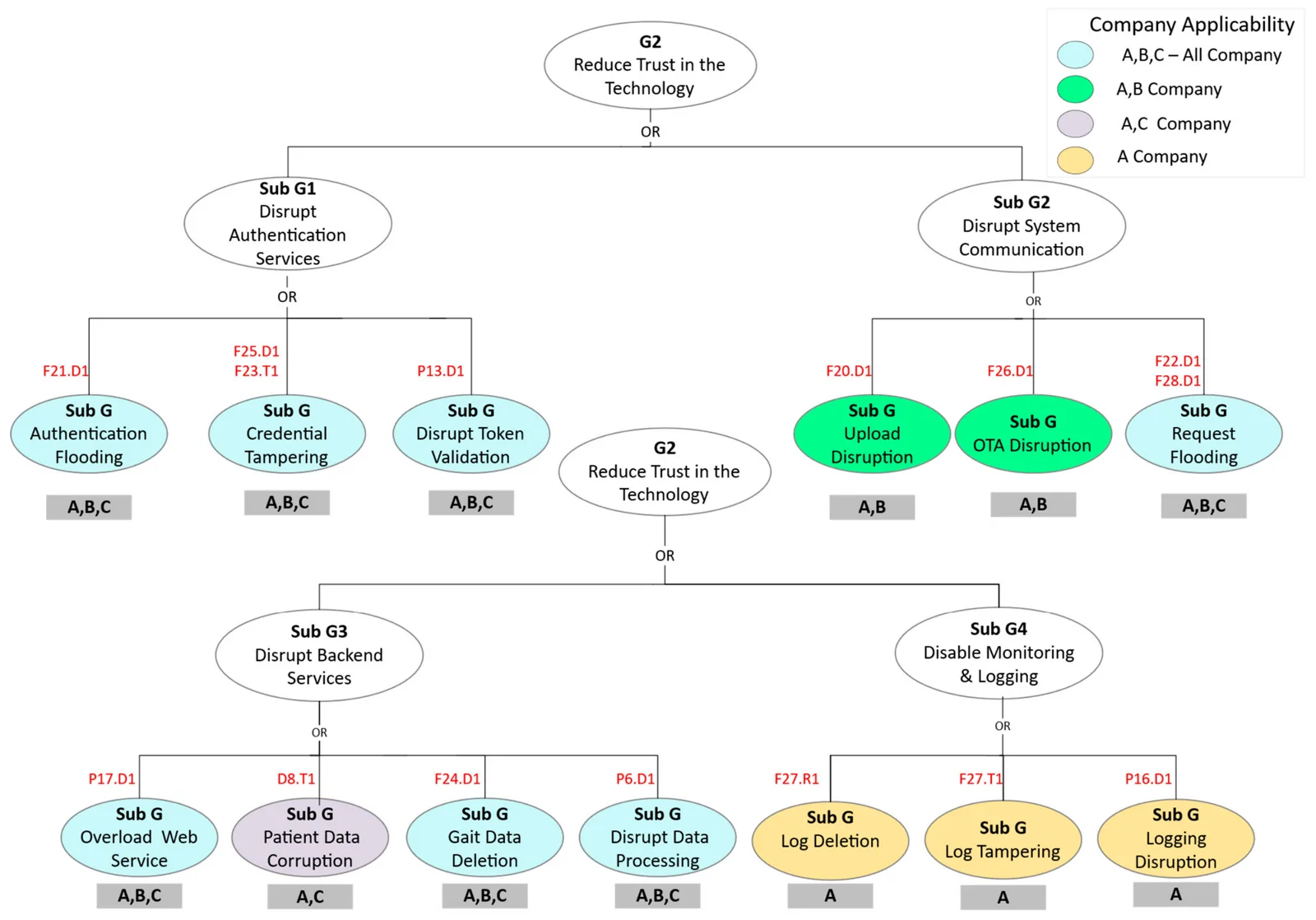
Attack tree for the sensing insole system representing attacker Goal 2: reducing trust in the technology. The tree illustrates how an adversary may combine authentication disruption (Sub G1), communication disruption (Sub G2), backend service disruption (Sub G3), and disabling of monitoring and logging (Sub G4) to undermine clinician and patient confidence in the system. Leaf node identifiers follow the notation defined in Figure 2; for example, F21.D1 denotes the first denial of service threat identified for data flow 21 in the STRIDE analysis.

**Figure 4:**
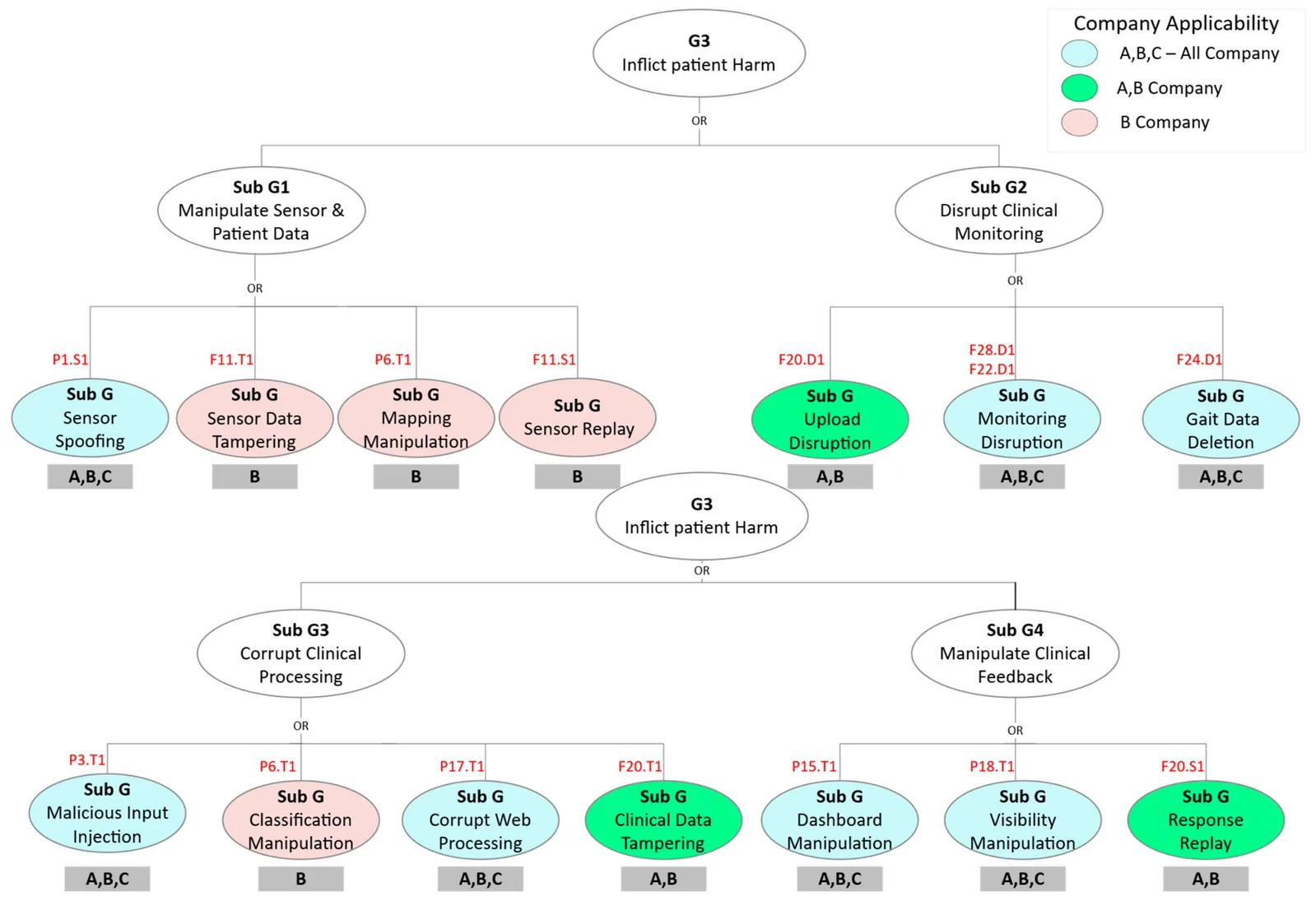
Attack tree for the sensing insole system representing attacker Goal 3: inflicting patient harm. The tree illustrates how an adversary may manipulate sensor and patient data (Sub G1), disrupt clinical monitoring (Sub G2), corrupt clinical processing (Sub G3), or manipulate clinical feedback (Sub G4) to cause harm to the patient through incorrect or disrupted clinical decision-making. Leaf node identifiers follow the notation defined in Figure 2; for example, P1.S1 denotes the first spoofing threat identified for process 1 (sensors) in the STRIDE analysis.

In this paper, we present the following contributions:

1. A data flow diagram representative of a clinical smart insole IoT system across four trust zones, derived from direct correspondence with several commercial manufacturers and publicly available information on these and similar systems (Figure 1).
2. A comprehensive STRIDE-based threat modelling analysis covering all system components, data flows, and data stores, producing a structured threat table (Table 1; Appendix A, Table A1a-d) for researchers and device manufacturers. Security threats were mapped against the MITRE ATT&CK framework to understand attacker techniques and tactics.
3. Three theoretical attacker-goal-driven (Table 2) attack trees (Figure 2, Figure 3 and Figure 4) representing realistic adversarial scenarios, cross-referenced to the STRIDE threat table.
4. A comparative modelled security analysis of three commercial smart insole systems mapped against the identified threats (Table 3).

The remainder of this paper is structured as follows:

The next section reviews related work. Section 3 describes the methodology, including the representative system architecture, threat modelling approach, and attack tree construction. Section 4, Section 5 and Section 6 report the modelled findings and discuss their implications. The full modelled STRIDE threat analysis and the complete threat table for the system are provided in Appendix A.

## 2. Related Work

The increasing adoption of medical IoT devices (also called Internet of Medical Things or IoMT devices [21]) in healthcare has introduced major cybersecurity issues. Unlike conventional IT systems housed in secure environments, medical IoT devices like wearable sensors and implanted monitors function wirelessly in unsecured areas that are difficult to protect [17]. This combination of broad connectivity and physical exposure makes them susceptible to multiple cybersecurity threats including database tampering, and data breaches. These can cause the leakage of sensitive patient information, disrupt clinical workflows, and erode trust between patients and healthcare providers [22].

Akinbi and Raj conducted a security analysis of medical IoT ecosystems across multiple threat-modelling scenarios, finding that spoofing and tampering were the most common threat categories and that integrity attacks receive less attention than confidentiality threats, despite their direct clinical consequences [17]. Severo et al. found that most threat modelling studies in healthcare focus on general hospital information systems or established device categories, with newer wearable sensing platforms receiving comparatively little attention [22]. Papaioannou et al. identified six essential security objectives for medical IoT systems: confidentiality, integrity, non-repudiation, authentication, authorisation, and availability, noting that integrity (ensuring data are not maliciously manipulated to alter device function) and availability (ensuring the device/system remains operational for the intended user) are particularly critical in clinical settings yet are often addressed less rigorously than confidentiality [21]. Hasan et al. similarly identified authentication weaknesses, data interception, and denial of service as primary concerns, highlighting that the absence of robust authentication in many medical IoT devices creates a persistent vulnerability [23]. Salayma argued that static threat models are insufficient for systems where adversarial behaviour evolves across interconnected components and trust boundaries, proposing dynamic attack graphs as a more suitable approach [24]. Bradbury et al. applied STRIDE-based threat modelling with attack trees to a resource-constrained IoT system, demonstrating how individual threats across system components can be chained into attack paths that support broader adversarial goals [12].

Several studies have shown cybersecurity vulnerabilities in connected digital medical devices. Alhussan et al. [16] developed a systematic auditing approach for investigating cybersecurity vulnerabilities of four different types of IoT medical devices including continuous glucose monitors and electrocardiogram (heart rhythm) monitors. This study demonstrated security vulnerabilities across all four IoT medical devices including successful man-in-the-middle attacks involving unauthorized data disclosure and manipulation. Marin et al. [15] and Li et al. [13] reported that several commercial insulin pumps failed to use any encryption in communicating between peripheral devices and were susceptible to unauthorized data disclosure and manipulation.

Although medical IoT device cybersecurity is an emerging research area, no previous study has utilised a structured threat modelling approach specifically for a smart insole system. Most analyses of wearable medical devices focus on the device itself or its communication link separately, without considering the entire data flow across different trust zones/boundaries. Furthermore, prior cybersecurity research has mainly concentrated on data confidentiality, with much less attention given to integrity and availability attacks, which could have far more damaging clinical consequences. This study fills these gaps by using a detailed data flow diagram (DFD)-based threat model on a smart insole system across four trust zones. It specifically examines integrity and availability threats that have direct clinical implications and compares the security strengths of three commercial products against the identified threats.

## 3. Methods

This section describes the methodology adopted in this study, covering the representative system architecture, the threat modelling approach, and the analytical frameworks used.

### 3.1. IoT Medical Insole Systems

This study focussed on a series of smart IoT medical devices known as smart sensing insole/sock systems, that provide the opportunity for remote patient monitoring. International devices include systems from several countries including the USA, Canada, UK, France, Germany and Ireland. The available smart sensing insole/sock IoT medical systems are similar in nature because many common parameters are measured across the systems and they have a similar health/medical function either predominantly focused on, or with implications for diabetes as a user/patient group. Key measurement parameters include foot pressure, foot temperature and gait (walking) parameters using accelerometers and gyroscopes. To understand system architectures, components and data flows in smart insole systems we worked with several companies producing smart insoles and used publicly available information and the scientific literature. Although there are several differences across the types of smart insole systems we worked with, we aimed to build a system architecture for this study that was broadly representative of these systems (and therefore not solely modelled/replicated on any one specific system). The system architecture developed is therefore a hypothetical architecture that we have determined to be broadly representative of these IoT medical systems, considering the key features of these systems to ensure relevance and validity. We used the modelled results and discussion of this manuscript to explore variations in smart insole system architecture, components and data flows between different specific devices for understanding their potential security and user implications, but we maintain confidential the details of the specific devices due to commercial and security sensitivities.

This study draws on two main sources of information. First, it provides a direct analysis of three commercial smart insole systems, detailing observed security features and configurations. These insights come from manufacturer documentation, publicly available technical data, the scientific literature and direct interactions with system developers. Second, it uses a synthesised reference architecture based on several smart insole systems to perform a systematic threat modelling using STRIDE, attack trees and MITRE ATT&CK mappings. Throughout the paper, outcomes related to the commercial systems are clearly separated from modelled threats identified through system architectural analysis. It is important to note that no penetration testing or other experimental vulnerability testing was carried out. All threats identified are based on threat modelling using the frameworks and approaches described.

### 3.2. Threat Model Approach

A threat model was adopted that assumed multiple adversaries with the following overall goals that have been demonstrated conceptually and/or practically feasible for similar digital healthcare technologies:

1. Steal user health data [13,16];
2. Reduce trust in the technology [15,16,25];
3. Inflict patient harm [13,16,26,27,28,29].

The malicious goals outlined above could be pursued by adversaries with financial, ideological or reputational motivations. This could include international state-supported adversaries, criminal groups and commercial competitors. To systematically identify and analyse the threats associated with these goals, this study adopted a framework-based threat modelling approach. Rather than relying on a single perspective, framework-based methodologies integrate multiple analytical dimensions, including attacker-centric, asset-centric, and data-centric views, into a cohesive, structured analysis [30]. The STRIDE framework was selected as the primary methodology for this study, supported by attack tree construction and MITRE ATT&CK mapping. This combination enables both systematic threat classification across system components and a structured representation of how individual threats combine into coherent attack paths towards each adversarial goal.

The methodology followed three sequential stages. First, a reference system architecture was developed by analysing the three commercial smart insole systems and synthesising their common components, data flows, and trust boundaries into a representative data flow diagram. Second, STRIDE threat modelling was applied systematically to each entity, process, data store, and data flow within the architecture, identifying and classifying potential threats across all six STRIDE categories. Third, the identified threats were mapped to attacker goals using attack trees, and further contextualised using the MITRE ATT&CK framework to identify relevant attacker tactics and techniques.

### 3.3. Threat Identification Using STRIDE

The STRIDE threat model is a comprehensive and structured framework approach that was used in the present study since it can be regarded as more adaptable to different contexts compared to other models and can be combined with other security methodologies to form a comprehensive approach to threat modelling. The STRIDE framework classifies cybersecurity threats into six types: Spoofing: an attacker impersonates another user, system or device to gain unauthorised access to a device, data or system with malicious intent. Tampering: unauthorised modification or manipulation of data, system components or physical elements with malicious intent. Repudiation: the unauthorised removal of audit trails or other forms of digital evidence that would otherwise enable tracking of malicious actions, allowing an attacker to deny accountability for their malicious activities. Information disclosure: unintended exposure of sensitive or confidential data to unauthorised individuals, resulting in data breaches and violations of privacy. Denial of service: an attacker makes a service, system or function unavailable to the intended legitimate users, resulting in disruption or cessation of normal operations. Elevation of privilege: an attacker gains higher access rights to a device or system than they are authorised for, allowing them to perform actions that should be restricted.

In this study, STRIDE was applied systematically across all components, data flows and trust boundaries defined in the reference architecture, given its suitability for structured threat identification in IoT and medical device contexts. It offers a repeatable approach to classifying threats across the six categories described above and sits alongside the attack tree and MITRE ATT&CK analyses as part of the broader threat modelling framework adopted here. The STRIDE framework was applied purely for identification and classification purposes; it does not assess threat likelihood, exploitability, attacker preconditions, or clinical severity, and was not intended to do so. Where certain threats are described as particularly significant, this reflects their potential clinical relevance to diabetic foot care rather than any formal risk assessment.

### 3.4. MITRE ATT&CK Mapping

The MITRE ATT&CK (Adversarial Tactics, Techniques, and Common Knowledge) knowledge base was used in this study to understand attacker techniques and tactics that could be applied to compromise the security of smart sensing IoT insole systems [31].

MITRE ATT&CK is a widely used cybersecurity knowledge base that documents adversary behaviours based on real-world attack observations. The framework organises attacker activities into tactics, which represent the objectives an attacker aims to achieve, and techniques, which describe the methods used to accomplish those objectives. By providing a structured representation of known attack behaviours, MITRE ATT&CK helps us to understand, analyse, and model potential attack scenarios. In this study, the threats identified through the STRIDE framework were mapped to specific attacker tactics and techniques using the MITRE ATT&CK framework, providing additional context on how security threats could be realised in practice and improving the traceability between system components, threats, and attacker behaviours. For example, threats involving data modification were mapped to T1565 (Data Manipulation), while denial-of-service-related threats were mapped to T1499 (Endpoint Denial of Service). It should be noted that these mappings represent analytical analogues derived from the threat modelling analysis and do not constitute evidence that the exact techniques have been observed or demonstrated in smart insole systems specifically.

### 3.5. Attack Tree

An attack tree provides a systematic theoretical approach to describing and analysing the security of a system against various types of attacker goals. In this study, attack trees were used to provide a hierarchical representation of attacker objectives, where the root node represents the attacker’s goal, intermediate nodes represent potential attack paths or sub-goals, and leaf nodes represent specific attack actions. The ‘OR’ nodes indicate alternative attack paths, where any ‘child’ node can satisfy the ‘parent’ objective. To maintain connectivity and traceability between the attack trees and other elements of the threat analysis, each leaf node was labelled using the corresponding STRIDE threat identifier, linking attack actions to the associated DFD component and identified threat.

### 3.6. System Architecture and Data Flow Diagram

Figure 1 presents the representative DFD of the smart insole/sock IoT medical system, based on a reference architecture, with component notation: processes (P), data storage (D), data flow (F), and external entities (E) defined in Section 4. The system is divided into four trust zones, each with a distinct role in data collection, processing, and communication, as described below.

### 3.7. External Entities

#### 3.7.1. Patient/User

The patient wears the smart insoles or sock system. The system collects various data from their feet and walking activity using embedded sensors.

#### 3.7.2. Clinician/Health Professional

The clinician remotely monitors their patient’s foot health and gait activity by accessing the collected data through a clinician dashboard. This allows remote monitoring of the patient’s condition and supports clinical decisions.

### 3.8. Sensor Zone

This zone within the smart insole or sock contains the main hardware and low-level software components. These include sensors (including pressure and temperature sensors, accelerometers and gyroscopes), a microcontroller processing unit (MCU), local storage, a Bluetooth Low Energy (BLE) module, secure key storage, and firmware storage. When the system is used for the first time, the insole connects to a mobile device using BLE. During this step, pairing is performed to establish a secure connection and generate cryptographic keys. Bonding then stores these keys in secure key storage (D2 in Figure 1) so that future connections are trusted. After setup, the sensors collect raw data. The processing unit extracts basic parameters such as walking speed and step length. Both raw data and processed parameters may be stored temporarily in local storage (D1 in Figure 1).

The insole sends data to the paired mobile device over BLE. This communication is encrypted and authenticated. The main data flow is from the insole to the mobile device, but control commands can also be sent back, for example, to change settings in the insole. The sensor zone also supports OTA firmware updates. The firmware is received from the mobile device via BLE and stored temporarily. Before installation, secure boot verifies the firmware. This ensures that the firmware is valid and has not been modified. The verification process depends on trusted keys stored in secure key storage.

### 3.9. Edge Zone

The edge zone (e.g., smartphone or home hub) acts as an intermediate layer between the insole/sock and the cloud. It manages communication, data processing, and user interaction. When the system starts, the edge zone handles user onboarding, account creation, and login. It manages BLE communication with the insoles, listening for incoming data and identifying which insole (left or right) sends the data, with each insole sending data independently. The mobile app/home hub receives these data streams and temporarily stores them in a buffer. The system waits until data from both insoles is available, and a matching process then aligns the data based on timestamps and device identity. After matching, the system merges the data into paired walking parameters. Only matched and processed parameters, such as walking speed and step length, are sent to the cloud. Raw sensor data is not uploaded.

The edge zone uses several storage components. A temporary buffer (D5 in Figure 1) stores incoming parameters before matching. Separate storage is used for user credentials, cryptographic tokens, and device configuration. The configuration storage keeps information such as device IDs, left and right insole mapping, BLE pairing data, and device settings. This information is required for correct data matching and system operation. Before sending data to the cloud, personal information is removed. Data are linked to a unique identifier instead of the patient’s name. This ensures that the cloud does not store direct personal identifiers.

Communication with the backend is performed through secure Application Programming Interface (API) calls. The mobile app/home hub uses authentication mechanisms such as API keys and user tokens. All data transfers are encrypted. The mobile application also manages user settings and system control. This process includes actions such as reconnecting devices, resetting pairing, updating device settings, and managing passwords. In addition, the system handles data visibility requests from users. These requests are processed, and decisions are stored locally or sent to the cloud. The structure supports secure communication, correct data processing, and controlled data sharing between the insole and the cloud.

### 3.10. Cloud Zone

The cloud zone contains the backend system and is responsible for data storage, processing, and access control. It includes API servers, a user management service, a clinical access layer, processing algorithms and a database. The backend receives processed data from the edge zone. It stores processed parameters derived from raw sensor data (insole zone), including walking speed and step length. Raw sensor data is not stored in the cloud. It also stores user account information required for system operation. All stored data is encrypted.

The backend provides APIs for communication with the mobile application and external users. User authentication and account management are handled through a dedicated user management service.

The clinical access layer manages data access requests from clinicians. When a clinician requests to view patient data, the system checks access permissions based on system policies. If the request is authorised, the system retrieves the required data from the database and returns it via the API. For security, the system uses a key management service (e.g., Azure Key Vault) to store and manage cryptographic keys, with backend services using these keys for secure operations. The backend includes an auditing mechanism for logging all actions, including data access, login attempts and password changes, supporting auditing and detection of cyber-attacks.

### 3.11. Healthcare Provider Zone

The healthcare provider zone represents how healthcare professionals interact with the system for remote patient monitoring of user foot health and gait data. Clinicians access patient data through an online dashboard system, which receives data collected from the insoles, including foot pressure, temperature and gait data. Data are processed in the backend and presented to the clinician in a clear format.

Access to the dashboard is secured using two-factor authentication (2FA). This adds an extra layer of protection during the login process and helps prevent unauthorised access. The clinician can review patient data to monitor foot health and gait data, identifying changes over time. Based on this information, the clinician can support patient care and make informed decisions.

The system supports communication of any health alerts or notifications with the patient, and the clinician can also monitor these alerts/notifications through the dashboard. Notifications or alerts, such as high foot pressure alerts, may be generated from the insole data and shared with the patient through the mobile application. The clinician can then monitor these alerts through the dashboard. Access to patient data is controlled, with the clinician only viewing data when permission is granted by the patient, and all access is managed through secure authentication and backend services.

## 4. Results

This section presents the modelled findings of the study, beginning with the representative data flow diagram, followed by the STRIDE threat analysis and attack trees for each attacker goal.

### 4.1. Data Flow Diagram

A data flow diagram (DFD) was modelled using external entities, processes, data storage, indications of data flow direction and trust boundaries (Figure 1). This was developed as a graphical representation of a hypothetical reference architecture based on several smart sensing insole systems (as described in the methods), showing the movement, storage and direction of data flow within the system and across trust boundaries/zones. The modelled system workflow starts with the sensor measurements collected by the smart insole, which are subsequently processed and transmitted to the mobile application via Bluetooth Low Energy (BLE), where device pairing, authentication, visibility management, data upload, and firmware update functions are performed. Patient gait data are subsequently uploaded to cloud storage and processing services, where authentication, access control, audit logging and patient data management are implemented. Authorised healthcare professionals can access patient information through clinical services, subject to authentication and visibility controls. The DFD also highlights critical trust boundaries, including BLE communication, internet-based communication between the mobile application and cloud services, and interactions between cloud services and healthcare providers, which form important areas for security analysis and threat identification.

It is worth noting that the reference architecture does not directly replicate any single commercial system, nor was it intended to. This was a deliberate decision, largely driven by commercial confidentiality and security sensitivities among the companies involved. The architecture is instead a synthesised design informed by several systems, built around their common features to ensure it remains broadly representative and valid. Table 3 sits alongside the DFD to contrast how specific security controls are implemented or not across three anonymised systems, grounding the reference architecture in real-world practice.

To facilitate cross-referencing across the DFD, STRIDE threat table, and attack trees, a unified identifier notation is employed throughout this study. Each component within the DFD is assigned a prefix that corresponds to its type: ‘P’ for processes (e.g., P17: Clinical Web Service), ‘F’ for data flows (e.g., F20: Data Upload), ‘D’ for data stores (e.g., D8: Anonymized Patient Data Storage) and ‘E’ for external entities (e.g., E2: Clinician). Threats identified for each component are subsequently labelled using the initial of the relevant STRIDE category followed by a numerical identifier (e.g., T1 for the first Tampering threat, S1 for Spoofing, D1 for Denial of Service, I1 for Information Disclosure, E1 for Elevation of Privilege, and R1 for Repudiation). Consequently, a threat identifier follows the format: [Component] [STRIDE category] [Number]; for instance, P17.T1 denotes the first Tampering threat targeting Process 17, while F20.D1 indicates the first Denial of Service threat affecting Data Flow 20. These identifiers are also utilised as labels for leaf nodes within the attack trees, illustrating which specific threats identified in the STRIDE analysis contribute to each attacker goal (see Appendix A Table A1a–d).

### 4.2. STRIDE Threat Modelling Framework

As shown in Table 1, the STRIDE framework identified potential modelled cybersecurity threats to the smart sensing insole system across six categories, mapped to their primary system impacts, affected components, and representative threats. It should be noted that the threats identified below are classified by category only and do not represent a formal assessment of likelihood or risk. A comprehensive threat analysis covering all entities, processes, data flows, and data stores, across and within the four trust zones is provided in Appendix A, Table A1. This STRIDE-based modelling analysis maps onto the attacker goals displayed in the attack trees (Figure 2, Figure 3 and Figure 4).

**Table 1:** High-level overview of cybersecurity analysis using the STRIDE framework for the sensing insole system. A comprehensive threat analysis of the insole system using the STRIDE framework is provided in Appendix A (Table A1).

STRIDE Category	Primary Impact on System	Example Components	Representative Threat
Spoofing	Establishes false device or user identity	P6, P12, P13, P14	Rogue Device Pairing, Credential Abuse
Tampering	Corrupts gait data integrity and clinical output	P3, P6, P17, F11, F20	Data Tampering, Replay Attack
Repudiation	Destroys audit trail enabling undetected attacks	P16, D10, F27	Log Deletion
Information Disclosure	Exposes sensitive patient and clinical data	D8, F11, F20, F25	Data Interception, Credential Extraction
Denial of Service	Disrupts authentication and data delivery services	P13, F21, F26	Request Flooding, Resource Exhaustion
Elevation of privilege	Bypasses access controls exposing restricted data	P12, P15, P17, P20	Privilege Escalation, Unauthorised Access

Tampering represents one of the most significant modelled threat categories identified in the analysis, affecting core processing components, including sensor data processing (P3), the matching and pairing algorithm (P6), the clinical web service (P17), and key data flows, particularly across trust boundaries: F11 and F20. Malicious exploitation of these security vulnerabilities could manipulate insole and gait data at multiple stages of the processing pipeline through data tampering or replay attacks, potentially compromising the integrity of clinical outputs and health alerts, supporting the attacker’s goal G3 (inflict patient harm; Figure 4).

Spoofing threats are concentrated at device pairing and authentication boundaries, with the matching and pairing algorithm in the edge zone (P6), app-side authentication (P12), cloud-side authentication (P13), and firmware update service (P14) identified as primary targets. Credential abuse and rogue device pairing could enable an attacker to impersonate legitimate users or devices, potentially granting unauthorised access to patient data and system resources, thereby supporting attacker goals G1 and G2 (steal user health data, Figure 2 and reduce trust in the technology, Figure 3, respectively).

Elevation of privilege threats were found to potentially affect several access control mechanisms across the edge and cloud zones, particularly the visibility management service (P15), the clinical web service (P17) and the patient data access process (P20). Successful exploitation of these potential security weaknesses could allow an attacker to bypass intended access restrictions and retrieve protected clinical information beyond their authorised scope, supporting attacker goals G1 and G2 (steal user health data, Figure 2, and reduce trust in the technology, Figure 3, respectively).

Information disclosure threats were primarily identified to be concentrated within the cloud zone data storage (D8) and data flows crossing BLE (insoles to edge zone) and internet (edge to cloud zone) trust boundaries, F11 and F20, respectively. These attack vectors expose sensitive patient and insole/gait data during transmission, while credential extraction targeting F25 within the cloud may further compromise the authentication infrastructure, directly supporting attacker goal G1 (steal user health data; Figure 2).

Repudiation threats, although affecting fewer components, carry significant implications for clinical accountability and regulatory compliance of the sensing insole system. The audit logging service (P16), audit log storage (D10), and associated log data flow F27 in the cloud zone, are potentially vulnerable to log deletion, rendering malicious activity untraceable and directly undermining the system’s ability to satisfy audit and compliance requirements. This supports attacker goal G2 (reduce trust in the technology, Figure 3).

Denial of service threats target authentication and device management functions, including cloud authentication (P13), authentication request data flows (F21), and firmware update commands (F26). Request flooding and resource exhaustion at these points could disrupt clinician access to patient records and interfere with legitimate device management operations, reducing overall system availability and supporting attacker goal G2 (reduce trust in the technology, Figure 3).

Overall, the Repudiation, Denial of Service, and Tampering categories contribute most directly to attacker goal G2 (reducing trust in the technology). To achieve this objective, an adversary does not necessarily need to steal data or cause direct physical harm. Instead, by exploiting weaknesses such as log deletion (P16, D10), authentication flooding (P13, F21), or persistent data tampering (P17, F20), an attacker can gradually undermine clinician and patient confidence in the system’s reliability and integrity through service disruption, record corruption, and the elimination of accountability mechanisms. Taken together, the identified threats span three adversarial goals: data theft (G1), trust reduction (G2), and patient harm (G3), reflecting the broad attack surface of a clinically deployed medical IoT system.

### 4.3. Attack Trees

Attack trees were used to map potential modelled threats to system components shown in the DFD (Figure 1) and developed for each high-level theoretical attacker goal to understand potential attack paths and specific actions/tactics to achieve these goals and compromise the system. Attack trees provide a structure to represent how an adversary might combine individual threats to achieve a broader objective: the root node representing the attacker’s goal, the child nodes representing alternative attack paths or intermediate sub-goals to reach the specific objective.

Figure 2 presents the attack tree for G1: Steal User Health Data, which impacts patient privacy directly since an adversary tries to obtain sensitive gait/insole and clinical data without authorisation. Three sub-goals are represented in the tree, each connected by an OR gate, representing a fundamentally different approach to the same outcome. Sub G1 represents access to sensitive health information directly from system services and storage locations. For this sub-goal, there are three possible attack paths: Unauthorised Access, API Exploitation, and Data Extraction. Unauthorised access combines two threats: bypassing visibility restrictions at the visibility management service (P15.E1) and sending a forged visibility request to the backend (F22.S2), which could allow an attacker unauthorised access to patient records. API exploitation targets the clinical web service with an injection attack (P17.T1) and a weak request validation vulnerability in the web request flow (F28.E1), allowing an attacker to manipulate backend logic or bypass access controls to retrieve restricted patient data. The last leaf node for sub-G1 is data extraction (D8.I1), which directly targets the anonymised patient data store, enabling the unauthorised retrieval of stored gait/insole data and clinical records bypassing the access control mechanisms.

Interception of communications (Sub G2) is the second subgoal concerning G1 but takes a completely different approach to the first subgoal (Figure 2). In contrast to the mechanism for Sub-G1 that focus on breaching stored data, Sub-G2 targets data in transit across trust boundaries between different system zones. This sub-goal has three branches connected by OR gates.

Pairing Interception (F13.I1) focuses on BLE pairing communications flow between the insole pairing process and the matching algorithm, capturing device and patient associated data as it crosses the BLE boundary. Data interception combines two flows of processed sensor data over BLE (F11.I1) and the gait/insole data upload to cloud storage (F20.I1), representing interception at two different points along the data path, one between the insole and edge zones and one at the internet boundary between the edge and cloud zones, respectively (Figure 2).

Firmware interception (F8.I1) targets firmware transfers between storage and update services, potentially exposing proprietary firmware images and implementation details that could be used in future attacks. While intercepted firmware does not directly contain patient data, it can be reverse engineered to identify vulnerabilities that allow for future data theft. This sub-goal is potentially significant because it does not require the attacker to break any access controls; if the data is insufficiently protected in transit, it can be captured passively without the system detecting anything unusual.

The third sub-goal is Compromise Authentication (Sub-G3; Figure 2). This sub-goal deals with attacks against authentication and credential management mechanisms. This sub-goal is especially important because if credentials, authentication tokens or session information are compromised, it could allow an attacker to impersonate a legitimate user and gain unauthorised access to sensitive patient data/information. This branch consists of four attack paths. One potential mechanism in this branch is Credential Abuse (P12.E1), which takes advantage of a weakness in the app-side authentication procedure to obtain unauthorised access without requiring any legitimate credentials at all. Extract Credentials (D7.I1; F25.I1) is concerned with credential information and not with the authentication process itself. The attacker can obtain the credentials from the local storage in the device (D7.I1) or intercept them during the verification on the cloud side (F25.I1). The stolen information can then be used to compromise the system access later.

The token interception threat involves stealing authentication tokens from the login request flow while it is moving between the mobile application/edge zone and the cloud. This allows the attacker to reuse a valid session without knowing the underlying credentials. In Request Replay (F21.S1), a previously captured authentication request is replayed to establish a new session with an old token. Without replay protection mechanisms, an attacker may be able to access the system using these tactics as a legitimate user without requiring valid credentials.

The most common STRIDE category for the three sub-goals is Information Disclosure (Figure 2). The attacker’s goal is to steal data instead of disrupting or manipulating the data. Elevation of Privilege threats (P15.E1; F28.E1) notably, are also found in Sub G1. As a result, in many real-world attacks, data theft requires gaining access that was not intended.

Figure 3 shows an attack tree with the root goal of reducing trust in technology. This is divided into four subgoals: disrupt the authentication service, disrupt system communication, disrupt the backend service, and disable monitoring and logging.

The first subgoal (Sub G1) focuses on authentication services, which are the entry point through which both patients and clinicians access the system. Three attack paths have been identified. Authentication Flooding (F21.D1) overloads the authentication request flow with too many requests, preventing legitimate users from successfully authenticating. Credential tampering (F25.D1/F23.T1) attacks credentials during storage or verification, potentially resulting in authentication failures or invalid user access attempts. Disrupt Token Validation (P13.D1) interferes with the cloud-based authentication service that validates authentication tokens. Altogether, these attacks can prevent legitimate users from accessing the system and undermine confidence in its reliability.

The second sub-goal, Disrupt System Communication (Sub-G2), focuses on communication flows that carry data between the edge, cloud, and clinical zones. Upload Disruption (F20.D1) interrupts the flow of insole/gait data from the mobile device/edge zone to cloud storage, cutting off the primary data path and leaving the clinical backend without current patient data. OTA Disruption (F26.D1) prevents firmware update commands from reaching the edge device, leaving the insole running potentially outdated or vulnerable firmware and impeding the system’s ability to patch security vulnerabilities remotely. Request flooding targets both the clinical web service (F28.D1) and the visibility request flow (F22.D1) at the same time, overloading communication channels and backend services with too many requests, degrading system responsiveness and affecting both patient and clinician interactions. These attacks lower the availability and dependability of the monitoring platform. These paths show how disrupting communication at various points in the system can potentially result in the same outcome: a clinician receiving no data, stale data, or no response at all.

The third subgoal of Disrupt Backend Services is related to backend services and cloud infrastructure where patient data is stored and processed. Overload Web Service (P17.D1) depletes resources within the clinical web service, limiting its ability to respond to legitimate requests. Patient Data Corruption (D8.T1) modifies stored patient information, which may affect clinical assessments and reduce trust in the system’s accuracy. Gait Data Deletion (F24.D1) deletes historical gait/insole records using the pruning flow, removing the longitudinal data required to track changes in a patient’s condition. While Disrupt Data Processing (P6.D1) impairs the system’s ability to correctly process and classify gait measurements. These attacks potentially directly affect the reliability and integrity of clinical services delivered by the platform.

The fourth subgoal, Disable Monitoring and Logging (Sub G4), addresses attacks that reduce system visibility and accountability. Log Deletion (F27.R1) deletes audit records that could be used to investigate malicious activities. Log Tampering (F27.T1) modifies log entries, raising concerns about the accuracy of recorded events. Logging Disruption (P16.D1) prevents the audit logging service from recording all security-related activities. While these attacks may not have an immediate impact on patient monitoring, they do undermine forensic investigation, incident response, and regulatory compliance, reducing trust in the system.

Finally, Figure 4 presents an attack tree for attacker goal 3, Inflict Patient Harm, structured around four subgoals. The first sub-goal, Manipulate Sensor and Patient Data (Sub-G1), is at the earliest stages of the pipeline before any clinical processing occurs (Figure 1). An attacker could theoretically achieve this in four different ways. The most direct is Sensor Spoofing (P1.S1), where false gait measurements are injected directly to the sensor before the data reaches the system. Sensor Data Tampering (F11.T1) targets the flow of processed sensor data between the processing component and insole metadata storage, corrupting gait/insole parameters as they cross the BLE boundary. Mapping Manipulation (P6.T1) uses a different approach where the attacker does not change the data itself but uses the matching and pairing algorithm to connect a device to the wrong patient, so that the gait/insole data of one patient will be assigned to another. Finally, Sensor Replay (F11.S1) sends previously captured gait data as if it were current, injecting stale measurements into the pipeline without the system being aware of any anomalies. All four of these pathways could lead to a user and/or clinician making decisions based on data that does not accurately reflect the patient’s condition.

Valid data availability for the clinician is the second sub-goal, which targets clinical monitoring functions. The Upload Disruption (F20.D1) prevents gait measurements from being uploaded and made available to clinicians in the cloud zone. Monitoring Disruption (F22.D1 and F28.D1) overloads or interferes with monitoring-related services, preventing clinicians from accessing patient records in a timely fashion. Gait Data Deletion (F24.D1) deletes stored gait/insole information required for longitudinal analysis and trend identification. Unlike integrity attacks, which alter information, these attacks seek to prevent information from becoming available when needed. In a clinical setting, interruptions to monitoring services could potentially delay interventions and reduce the effectiveness of remote patient management.

The third sub-goal explores the system more deeply and concerns the cloud-side components that transform gait data into clinical outputs. Malicious Input Injection (P3.T1): Malicious inputs are injected into the sensor processing component, which affects subsequent analytical results. Classification Manipulation (P6.T1) again attacks the matching and pairing algorithm, but this time to generate incorrect gait classifications instead of incorrect device associations. Corrupt Web Processing (P17.T1) attacks the clinical web service using injection-based attacks that alter the application behaviour or processing logic. Clinical Data Tampering (F20.T1) alters data at upload, affecting what is ultimately stored and displayed.

The last branch of this attack tree is ‘Manipulate Clinical Feedback’ and is directed at the presentation layer, i.e., what the clinician sees rather than how data is collected or processed. The visibility management service is used to manipulate the data being displayed on the dashboard (P15.T1). A clinician may be looking at altered or fabricated patient information without realising it. Visibility Manipulation (P18.T1) directly attacks the visibility request process, dictating what data is presented to which clinician, possibly hiding important gait anomalies completely (F20.S1) utilises previously obtained responses or uploads, presenting old patient data as current data These attacks do not directly alter the underlying records that are stored but do affect the information available to healthcare professionals and so can potentially influence clinical judgement. Tampering is the most common threat category among the four sub-goals, which is consistent with the overall STRIDE modelling analysis and reflects the system’s particular vulnerability to integrity attacks at various points in the clinical pipeline.

Three theoretical attacker goals were identified for the sensing insole system, each linked to a different type of attacker, motivation, and required skills, as summarized in Table 2. The first goal is to steal user health data, often targeted by external attackers or organized cybercrime groups for money or identity theft. The second goal aims to undermine system trust, with competitors or hacktivists trying to disrupt the system or damage reputation without needing to access patient data. The third goal is to cause harm to patients, which has the most serious health consequences and requires being within a given distance of the device.

**Table 2:** Attacker goal profiles in the smart insole threat model: characterising threat actors, motivations, requirements, tactics, and the primary security impacts of each adversarial goal.

Goal	Threat Actor	Motivation	Requirements	Tactics	Primary Security Impact
**G1:** **Steal User Health Data**	External attacker, malicious insider, organised cybercrime group, hostile state.	Financial gain; identity theft; Targeted patient data theft	BLE/cloud access; credential or token interception; API knowledge	Eavesdropping; credential theft; replay attacks	Loss of confidentiality and patient privacy
**G2:** **Reduce Trust in the Technology**	Competitor organisation; actor; hacktivist group, hostile state.	Competitive disruption; sabotage; reputational damage	Access to authentication or upload services; request flooding capability	Service disruption; log tampering; persistent attacks	Reduced availability and user confidence
**G3:** **Inflict Patient Harm**	Malicious insider; sophisticated external **attacker**, hostile state.	Harm patients; manipulate clinical outcomes; exploitation of device vulnerabilities	BLE proximity; access to cloud processing; knowledge of clinical workflows	Sensor manipulation; device-patient mapping attacks; alert suppression	Compromised data integrity and patient safety

Table 3 compares the security features used in three commercial smart insole systems, denoted as Devices A, B, and C. The comparison includes some security aspects of the device, communication and cloud layers. Device-A is the only product that uses BLE Secure Connections and single-device bonding, providing the most robust protection at the wireless communication layer; however, its reliance on a smartphone as the sole gateway introduces a dependency on a consumer device that is not directly controlled by the system. Device-B provides the least protection at the BLE layer as data are transmitted in cleartext, with no device level encryption and partial cloud side encryption, making it particularly vulnerable to the interception threats described at F11 and F20 in the threat model. Device-C takes a different approach, using encryption at the application layer within the firmware rather than at the BLE link layer, and routing clinical data through a dedicated cellular hub rather than a smartphone, which significantly reduces gateway dependency and provides the strongest end-to-end encryption posture of the three devices. Both Devices A and C provide two-factor authentication and role-based access controls for clinician access, while only Device-B uses basic authentication and represents a meaningful gap in credential protection. Section 5 discusses in detail how these differences relate to the identified threats and their implications for the design of secure clinical IoT systems.

**Table 3:** Comparison of security features across sensing insole systems from different company devices.

Security Aspect	Device-A	Device-B	Device-C
BLE Secure Connection	Yes	No	No
Gateway Design	Smartphone	Smartphone	Dedicated Cellular Hub
Dependency on User Device	High	High	Low
Data encryption layer	Channel-level	Smartphone only	Application-level
Data Exposure over BLE	Protected	Cleartext	Encrypted Payload
End-to-end encryption	Partial	No	Yes
Single-device bonding	Yes	No	N/A
At-rest encryption (cloud)	Yes	Partial	Yes
Clinician Access Control	Yes (2FA, role-based)	Yes (Basic)	Yes (2FA, role-based)
Data anonymization	Yes	Partial	N/A
Dedicated cellular gateway	No	No	Yes
Local data storage	Yes	No	Yes

## 5. Discussion

This is the first study to undertake a comprehensive cybersecurity threat modelling analysis for a classification of medical IoT systems known as smart sensing insole systems. These smart sensing insole systems have applications for managing lower limb health by monitoring foot health and gait across several medical conditions. They are particularly useful in diabetes for preventing foot wounds known as diabetic foot ulcers that frequently lead to limb amputation. As a result, these medical IoT systems are proliferating across health services internationally and represent a positive development for the treatment and management of people with diabetes and other chronic diseases. From a cybersecurity perspective, these sensing insole systems remain unexplored, while other similar medical IoT technologies have demonstrated several areas of cybersecurity vulnerabilities in large part due to their resource-constrained nature and OTA communications [13].

The identified theoretical threats are potentially significant because they extend beyond typical data security issues. In diabetic foot care, smart sensing insoles monitor plantar pressure constantly to identify high-pressure episodes, which can lead to foot ulcers. If an attacker interrupts this monitoring by blocking data uploads, overwhelming the web service with requests, or deleting pressure records, the patient does not receive important alerts about high foot pressure. Healthcare professionals also cannot remotely monitor the patient’s foot pressure. In theory, this not only causes system downtime but also misses an important opportunity for intervention, during which high pressures might result in foot ulcer(s), infection and even the need for amputation. This example shows how threats like Denial of Service or Tampering can potentially cause real harm to patients and why it is important to consider not just data confidentiality, but also the availability and integrity of these systems.

A detailed reference/hypothetical system architecture was developed for the sensing insole system to understand the system’s operating zones, processes and data stores, the interaction of entities with the system and the data flows within and between zones (Figure 1). The established STRIDE framework was used to model and support classification of cybersecurity threats and attack trees to represent theoretical attack paths and mechanisms to achieve specific attacker goals. The MITRE ATT&CK framework was used to map modelled cybersecurity threats against the relevant classifications to provide further insight to attacker techniques and tactics. Although previous research has highlighted vulnerabilities associated with other medical IoT systems including continuous glucose monitoring systems, insulin pumps and electrocardiogram monitors [13,14,15,16,17] this study is novel in uncovering potential cybersecurity vulnerabilities for sensing insole systems.

This study highlighted potential cybersecurity threats across all six categories of the STRIDE framework (Table 1, Table 2, Table 3 and Table A1), with Tampering representing one of the most significant threats affecting areas including core processing components and data flow within and across trust boundaries. These cybersecurity threats theoretically have the potential to inflict patient harm through interruption of high-pressure alert remote monitoring. The sensing insoles are used for remote monitoring of diabetic foot care by tracking plantar foot pressure to identify prolonged high-pressure episodes, which are recognized as precursors to the development of diabetic foot ulcers. If an attacker disrupts this monitoring pathway, whether by blocking gait data uploads, flooding the clinical web service with requests, or deleting historical pressure records via the pruning flow, the patient may not receive any crucial high foot pressure alerts that would otherwise enable them to act upon this to change their foot loading behaviour and avoid a diabetic foot ulcer. In addition, the clinician would lose the ability to remotely monitor patient foot pressures. The potential consequence is not merely a loss of system availability but a missed clinical opportunity for patient and/or clinician intervention, during which undetected high foot pressures could lead to development of a diabetic foot ulcer, that could in turn lead to infection and the need for lower limb amputation. This theoretically illustrates how cyber threats classified as Denial of Service or Tampering can translate into real-world patient harm and underlines why this analysis deliberately extended beyond data confidentiality to examine availability and integrity threats in detail. Although this clinical scenario is plausible, the present approach does not quantify the risk or probability of this clinical risk and remains to be determined by future research.

Attack trees highlighted that stealing user health data could potentially be achieved through three main attack paths by accessing patient data, intercepting communications and compromising authentication (Figure 2). Patient data within the cloud zone can be accessed via several vectors (visibility layer, API or storage), which means that no single mitigation measure would be sufficient to protect unauthorised information disclosure in isolation. Interception of communications across trust boundaries between different system zones includes data crossing the BLE boundary (crossing insole and edge zones) and the internet boundary (crossing edge and cloud zones). If data are transmitted unencrypted as we found for one company device (Table 3), and as previously demonstrated for several insulin pumps [15], the lack of encryption represents a key vulnerability. Firmware interception is a particularly interesting vulnerability uncovered in the present study since it does not contain patient data but allows for future data theft without specifically breaching access controls. System authentication can be compromised through several mechanisms, but the structure of this attack tree underlines a key point: that an attacker could potentially intercept credentials at the edge zone, replay them at the cloud boundary and then extract patient records directly from storage, with each step building on the previous.

Analysis showed the attacker goal of reducing trust in the technology could be achieved through several mechanisms including disrupting system availability, communications, backend services and disabling logging capabilities required for audit. These attacks cover data flows across and within all system zones and primarily involves denial of service and tampering, in line with findings from other IoT medical devices including continuous glucose monitors and electrocardiogram (heart rhythm) monitors [16]. Our threat modelling results show that an attacker can inflict patient harm via the sensing insole system by manipulation of how data are collected, processed and by controlling data availability to the patient and healthcare professional. Maliciously altering data or disrupting the availability of valid data for user and clinical decision-making can compromise the effectiveness of remote patient monitoring and delay any necessary clinical interventions. Importantly, none of these attacks theoretically require physical access to the device and can be conducted OTA within a given proximity to the insole device (insole and edge zones), or remotely via the cloud zone. Proximity of successful OTA security attacks for other IoT medical devices has been reported at ~20 metres [13] or even up to ~100 metres depending upon signal and receiver strength [5].

The variations in security strategies among the three commercial systems studied are driven more by design choices and risk management priorities than by technical limitations. Devices A and B utilize the patient’s smartphone as the main interface, which lowers manufacturing costs but places much of the security obligation on a consumer device outside the control of manufacturers or clinicians (Table 3). Device C employs a dedicated cellular hub, eliminating this dependency but reducing user control over system functions. The lack of BLE encryption in System B and the absence of two-factor authentication for clinicians likely do not stem from a lack of awareness, as these security measures are common and well-known. This indicates that the differences in security levels among the three commercial smart insole products examined in this study may be due to prioritization rather than knowledge gaps, and such issues could be addressed through regulatory and procurement standards.

Based on the threat modelling analysis (Table 1 and Table 2; Figure 1, Figure 2 and Figure 3) and the three devices compared in the present study (Table 3), these findings carry important implications for designing future smart insole systems. First, BLE-layer security should be a fundamental requirement due to the demonstrated feasibility of interception at clinically relevant distances. Second, relying solely on password authentication is a systemic vulnerability, as attack tree analysis for Goal 1 shows how credential interception at the edge zone, replay at the cloud boundary, and data extraction from storage can form a continuous attack chain, with each step leveraging the previous one. Third, implementing audit logging should be a standard design feature rather than an optional aspect left to individual manufacturers, since lacking comprehensive logs hampers post-incident investigation and regulatory compliance. All identified attacks can be performed OTA without physical device access, either nearby or remotely via the cloud. Participating commercial organizations were anonymized as Device-A, Device-B, and Device-C for security and commercial sensitivity. Data were collected from publicly available information and communications with company representatives. The three systems compared in Table 3 were selected as they represent well-established and prominent commercial systems within the smart sensing insole market. Manufacturers of these systems were engaged directly by the research team, providing architectural and security feature information that forms the basis of the comparisons presented in Table 3. Device comparisons presented in Table 3 are limited to the three specific devices considered, although we believe that this provides a useful indication of smart insole system variation and security implications.

It should be noted that the research presented in this paper is based on modelling cybersecurity threats to the smart sensing insole system and not based on experimental laboratory testing these vulnerabilities through penetration testing or other experimental methods. As such, modelled findings from this study remain to be confirmed through experimental testing. The threat modelling analysis, however, was very extensive covering 20 processes, 10 data stores, 34 data flows, and 2 external entities across four trust zones, producing 219 modelled threats across six STRIDE categories. The construction of the reference architecture and systematic application of STRIDE required substantial expert time and domain knowledge, which may present a practical challenge for smaller healthcare organisations with limited cybersecurity resources.

Overall, this study highlights the importance of early application of structured threat modelling to specific medical IoT devices, preventing insecure designs from becoming entrenched after deployment. The DFD and threat model provided here offer a reusable framework for manufacturers, clinicians and regulators to evaluate security in sensing insole systems and similar wearables.

## 6. Conclusions and Future Works

Smart sensing insole systems monitor foot and lower limb health across several medical conditions, in particular diabetes. This study highlighted potential cybersecurity threats across all six categories of the STRIDE framework with tampering representing one of the most significant potential threats. The analysis identified 219 modelled threats spanning all system components and trust zones. Three attacker goals were modelled: stealing user health data, reducing trust in the technology, and inflicting patient harm, each supported by a corresponding attack tree and mapped to relevant MITRE ATT&CK techniques. Analysis of three commercial systems also revealed notable variation in key security controls including BLE encryption, authentication mechanisms and audit logging.

Cybersecurity threats on smart sensing insole systems theoretically extend beyond typical data security issues and have the potential to inflict patient harm through interruption of high-pressure alert remote monitoring without requiring physical access to the device. Future research using experimental methods including penetration testing is required to confirm our modelling results presented here. Further work should consider development of a qualitative risk matrix to prioritise threats according to their likelihood and clinical severity.

## Data Availability

The datasets presented in this article are not readily available due to security and commercial sensitivity. Requests to access the datasets should be directed to Neil Reeves.

## Appendix A

**Table A1:** Comprehensive cybersecurity analysis for the smart sensing insole system using the STRIDE framework and focussing on a: External Entities, b: Processes, c: Data Stores and d: Internal and Cross Data Flows.

**(a) External Entities**					
**Component/** **Communication Flow**	**Attacker Goal**	**Threat**	**STRIDE**	**MITRE ID**	**Description**
E1: Patient	Impersonate legitimate patient	Credential Abuse	Spoofing	T1078	Stolen or fabricated patient credentials are used to gain unauthorised access to the mobile application.
	Inject malicious input into the system	Malicious Input Injection	Tampering	T1565	Crafted or manipulated input data is submitted through the patient interface to alter system behaviour or corrupt processing.
	Access unauthorized patient records	Authorization Bypass	Information Disclosure	T1005	Weak access-control mechanisms allow access to data belonging to other patients
E2: Clinician	Impersonate clinician to access patient records	Credential Abuse	Spoofing	T1078	Compromised clinician credentials are used to access restricted patient information.
	Access records beyond assigned permissions	Privilege Escalation	Elevation of Privilege	T1068	Excessive or misconfigured privileges allow unauthorised access to additional patient records.
	Extract bulk patient records	Data Exfiltration	Information Disclosure	T1005	Legitimate clinician access is abused to export sensitive patient information.
F1: Sensor Read (E1 → P1)	Intercept raw gait measurements	Data Interception	Information Disclosure	T1040	Raw sensor measurements are intercepted before entering the sensor processing pipeline
	Inject falsified sensor readings	Sensor Data Tampering	Tampering	T1565	Manipulated sensor measurements are injected into the processing workflow.
F14: Change App Setting (E1 ↔ P10)	Modify application behaviour	Configuration Tampering	Tampering	T1565	Application settings are modified to alter system behaviour or security controls.
	Bypass configuration restrictions	Unauthorized Configuration Change	Elevation of Privilege	T1078	Configuration settings are abused to bypass intended restrictions.
F15: Login Credential (E1 → P12)	Capture patient credentials	Credential Interception	Information Disclosure	T1552	Patient credentials are intercepted during authentication.
	Reuse valid credentials	Replay Attack	Spoofing	T1550	Previously captured authentication credentials are replayed to gain unauthorised access.
	Prevent legitimate authentication	Authentication Flooding	DoS	T1499	Excessive authentication attempts disrupt login services.
F16: Visibility Request (E1 ↔ P11)	Submit forged visibility requests	Request Spoofing	Spoofing	T1078	Forged visibility requests are submitted to gain unauthorised access to patient information.
	Manipulate visibility decisions	Visibility Manipulation	Tampering	T1565	Visibility permissions are modified to expose or hide patient data.
	Prevent visibility decision processing	Request Flooding	DoS	T1499	Excessive visibility requests disrupt processing of legitimate patient consent decisions.
F32 Access Patient Data (E2 ↔ P20)	Unauthorized Access to patient records	Request Spoofing	Spoofing	T1078	Forged clinician requests are submitted to gain unauthorized access to patient records
	Modify returned patient information	Request Tampering	Tampering	T1565	Request parameters are manipulated to alter returned patient data
	Intercept patient data in transit	Data Interception	Information Disclosure	T1040	Patient data is intercepted during clinician-system communication.
F33: Login Credential (E2 → P19)	Capture clinician authentication token	Token Interception	Information Disclosure	T1552	Authentication tokens are intercepted during clinician login
	Reuse valid authentication token	Replay Attack	Spoofing	T1550	Previously captured authentication tokens are replayed to gain unauthorized access.
	Modify authentication response	Token Tampering	Tampering	T1565	Authentication tokens or responses are altered to escalate privileges.
F34 Visibility request (E2 ↔ P18)	Access restricted visibility information	Request Spoofing	Spoofing	T1078	Forged visibility requests are submitted using compromised clinician credentials.
	Modify visibility decisions	Visibility Decision Tampering	Tampering	T1565	Visibility responses are modified before delivery to clinician systems.
	Replay previous visibility response	Replay Attack	Spoofing	T1550	Previously valid visibility responses are resent to bypass restrictions.
**(b) Processes**					
**Component** **/Communication Flow**	**Attacker Goal**	**Threat**	**STRIDE**	**MITRE ID**	**Description**
Sensor (P1)	Inject false gait measurements	Sensor Spoofing	Spoofing	T1036	Fake/malicious data injected to sensors
	Modify raw sensor values	Sensor Signal Tampering	Tampering	T1565	Sensor measurement data are modified before processing.
Insole Pairing (P2)	Pair unauthorized device	Authentication Bypass	Spoofing	T1550	An unauthorized device bypasses pairing verification.
	Reuse valid pairing session	Replay Attack	Spoofing	T1550	Captured pairing exchange is replayed.
	Disrupt pairing process	Request Flooding	DoS	T1499	Excessive pairing requests block legitimate pairing.
Sensor Processing (P3)	Alter processing results	Data Tampering	Tampering	T1565	Processed gait data is modified before output.
	Malicious Input Injection	Malicious Input Injection	Tampering	T1190	Manipulated sensor data is injected into the processing pipeline to influence gait analysis results.
	Exhaust processing resources	Resource Exhaustion	DoS	T1499	Excessive inputs disrupt legitimate processing operations.
Secure Boot (P4)	Execute unauthorized firmware	Firmware integrity bypass	Tampering	T1553	Secure boot verification is bypassed to execute malicious firmware.
	Trust malicious firmware	Key Substitution	Tampering	T1565	Attacker replaces legitimate verification keys with counterfeits
Firmware Installer (P5)	Disrupt firmware update process	Update Service Disruption	DoS	T1499	Over-the-air (OTA) delivery is disrupted, preventing legitimate updates.
	Install malicious firmware	Command Spoofing	Spoofing	T1550	Forged OTA commands trigger malicious firmware installation.
	Install vulnerable firmware	Firmware Downgrade	Tampering	T1601	Older vulnerable firmware is replayed or reinstalled.
	Modify firmware package	Firmware Tampering	Tampering	T1565	Firmware packages are altered before installation.
Insoles Matching & Pairing Algorithm (P6)	Alter matching decisions	Data Tampering	Tampering	T1565	Insoles pairing logic is modified to produce false matches.
	Associate rogue device	Unauthorized Device Association	Spoofing	T1550	Unauthorized device is matched and paired.
	Disrupt pairing operations	Resource Exhaustion	DoS	T1499	Excessive pairing requests disrupt legitimate pairing operation.
Data Upload (P7)	Modify uploaded gait data	Data Tampering	Tampering	T1565	Gait data is modified before being uploaded to the cloud
	Reuse old patient data to influence processing results	Replay attack	Tampering	T1550	Previously captured patient data is resent to backend services.
	Intercept patient data	Data interception	Information Disclosure	T1040	Sensitive gait data is intercepted during transmission.
	Prevent cloud data delivery	Upload disruption	DoS	T1499	Data upload is disrupted before reaching cloud services.
Firmware Update (P8)	Trigger unauthorized firmware installation	Command Spoofing	Spoofing	T1550	Unauthorized OTA commands trigger spurious firmware updates.
	Reinstall vulnerable firmware	Replay Attack	Spoofing	T1550	Previously valid update commands are replayed to compromise firmware security.
	Disrupt firmware update process	Request Flooding	DoS	T1499	Excessive update requests disrupt OTA operations.
Prune History (P9)	Remove historical data records	Data Deletion	Tampering	T1565	Historical gait records are removed from storage compromising data integrity.
	Conceal clinically significant data	Pruning Rule Tampering	Tampering	T1565	Pruning rules are modified to conceal clinically significant foot pressure/gait data.
App Settings Management (P10)	Modify application settings	Configuration Tampering	Tampering	T1565	Application settings are modified illegitimately to alter system functionality.
	Bypass configuration restrictions	Unauthorized Configuration Change	Elevation of Privilege	T1078	Unauthorized user modifies application settings without proper access rights.
	Disable application controls	Configuration Disruption	DoS	T1499	Application settings are altered to disrupt system operation.
	Expose restricted application data	Visibility Manipulation	Tampering	T1565	Visibility-related settings are modified improperly.
Visibility Management (P11)	Expose restricted patient data	Visibility Manipulation	Tampering	T1565	Visibility settings are altered, exposing or hiding data inappropriately.
	Access unauthorized patient records	Unauthorized Data Access	Information Disclosure	T1005	Restricted patient data is accessed without authorization.
	Bypass visibility restrictions	Authorization Bypass	Elevation of Privilege	T1068	Access restrictions are bypassed to retrieve restricted data
	Disrupt visibility processing	Request Flooding	DoS	T1499	Excessive visibility requests disrupt processing services.
App-Side Authentication (P12)	Access system without valid credentials	Authentication Bypass	Elevation of Privilege	T1068	Attacker bypasses authentication checks.
	Use stolen patient credentials	Credential abuse	Spoofing	T1078	Stolen credentials are used for unauthorized access.
	Reuse valid authentication session	Replay Attack	Spoofing	T1550	Captured authentication tokens are reused by an unauthorized user.
	Use stolen login credentials to gain account access	Credential Stuffing	Spoofing	T1110	Automated login attempts using stolen credentials are used to gain unauthorized account access.
	Disrupt authentication service	Authentication Flooding	DoS	T1499	Excessive authentication requests disrupt login services.
Cloud-Side Authentication (P13)	Access backend services without authorization	Authentication Bypass	Elevation of Privilege	T1068	Unauthorized attacker bypasses cloud authentication controls.
	Use stolen credentials or session tokens	Credential Abuse	Spoofing	T1078/T1550	Valid credentials or tokens are abused to enable unauthorized access.
	Reuse valid authentication session	Replay Attack	Spoofing	T1550	Previously captured authentication tokens are replayed to gain unauthorized access.
	Use stolen login credentials to gain cloud access	Credential Stuffing	Spoofing	T1110	Automated login attempts using stolen credentials are used to gain unauthorized cloud access.
	Disrupt authentication availability	Authentication Flooding	DoS	T1499	Excessive authentication requests disrupt cloud login authentication services.
Firmware Update Service (P14)	Send unauthorized firmware updates	Command Spoofing	Spoofing	T1550	Forged OTA commands trigger unauthorized firmware updates.
	Modify firmware update package	Firmware Tampering	Tampering	T1565	Firmware packages are modified before delivery to compromise security.
	Install vulnerable firmware version	Firmware Downgrade	Tampering	T1601	Older, more vulnerable firmware is redistributed.
	Disrupt firmware delivery service	Update Service Disruption	DoS	T1499	OTA update delivery is disrupted to reduce security.
Visibility Management (P15)	Access restricted patient records	Unauthorized Data Access	Information Disclosure	T1005	Restricted patient data is exposed without authorization.
	Alter data visible to clinician	Data Presentation Manipulation	Tampering	T1565	Data displayed to the clinician is maliciously manipulated.
	Disrupt visibility authorization service	Request Flooding	DoS	T1499	Excessive requests disrupt visibility processing.
	Bypass visibility restrictions	Authorization Bypass	Elevation of Privilege	T1068	An unauthorized user expands access to visibility controls.
Audit Logging Service (P16)	Modify security audit records	Log Tampering	Tampering	T1565	Audit records are modified to hide malicious activity.
	Disrupt logging operations	Logging Disruption	DoS	T1499	Logging services are overwhelmed by excessive requests, preventing audit events from being recorded.
	Access sensitive audit information	Unauthorized Log Access	Information Disclosure	T1005	Audit records are accessed without authorization.
	Delete audit trails	Log Deletion	Repudiation	T1070	Security logs/audit trails are deleted to remove evidence of any malicious actions.
Clinical Web Service (P17)	Escalate service privileges	Privilege Escalation	Elevation of Privilege	T1068	Attacker gains unauthorized privilege expansion.
	Hijack clinician session	Session Hijacking	Spoofing	T1550	Stolen session tokens are reused for access to the clinician dashboard.
	Manipulate backend processing	Injection attack	Tampering	T1190	Unauthorized manipulation of backend application processing.
	Access restricted patient information	Unauthorized Data Access	Information Disclosure	T1005	Attacker gains unauthorized access to backend services exposing patient data.
	Disrupt clinician service access	Service Disruption	DoS	T1499	Excessive requests maliciously disrupt web service availability.
Visibility Request (P18)	Submit forged visibility requests	Request Spoofing	Spoofing	T1078	Forged requests are submitted using compromised identities.
	Bypass visibility approval controls	Authorization bypass	Elevation of Privilege	T1068	Approval mechanisms are bypassed to gain unauthorized access.
	Modify visibility request decisions	Visibility Manipulation	Tampering	T1565	Visibility requests or responses are maliciously altered.
	Disrupt visibility request processing	Request Flooding	DoS	T1499	Excessive requests disrupt visibility operations.
	Access restricted visibility information	Unauthorized Data Access	Information Disclosure	T1005	Visibility-related patient data is exposed to unauthorized user.
Clinician Login (P19)	Hijack active clinician session	Session Hijacking	Spoofing	T1550	Active session tokens are reused for unauthorized access.
	Gain access with stolen credentials	Credential Abuse	Spoofing	T1078	Stolen clinician credentials are used to gain unauthorized access.
	Access clinician dashboard without authorization	Authentication Bypass	Elevation of Privilege	T1068	Dashboard authentication controls are bypassed by unauthorized user.
	Expose clinician or patient information	Unauthorized Data Access	Information Disclosure	T1005	Sensitive patient data is exposed to an unauthorized user.
	Disrupt clinician dashboard availability	Authentication Flooding	DoS	T1499	Excessive malicious login requests disrupt clinician dashboard access.
Access Patient Data (P20)	Impersonate authorized user	Credential Abuse	Spoofing	T1078	Attacker uses stolen credentials to gain unauthorized access to patient data.
	Access restricted patient records	Unauthorized Data Access	Information Disclosure	T1005	Restricted patient records are accessed by an unauthorized user.
	Modify retrieved patient information	Data Tampering	Tampering	T1565	Returned patient data is maliciously altered before use.
	Bypass patient-access restrictions	Authorization Bypass	Elevation of Privilege	T1068	Access controls are bypassed to retrieve patient data/records.
	Disrupt patient data retrieval	Service Disruption	DoS	T1499	Excessive requests disrupt patient-data access services.
**(c) Data Store**					
**Component/** **Communication Flow**	**Attacker Goal**	**Threat**	**STRIDE**	**MITRE ID**	**Description**
Sensor Data Storage (D1)	Modify stored insole/gait measurements	Data Tampering	Tampering	T1565	Stored sensor data is maliciously altered before processing.
	Access stored sensor measurements	Unauthorized Data Access	Information Disclosure	T1005	Stored sensor data is accessed without authorisation.
	Delete stored sensor history	Data Deletion	Tampering	T1565	Unauthorised removal of stored sensor records/data.
Secure Key Storage (D2)	Disable access to secure keys	Key Storage Corruption	DoS	T1499	Secure key storage becomes unavailable or corrupted.
	Access stored cryptographic keys	Key Extraction	Information Disclosure	T1552	Cryptographic keys are extracted from secure storage.
	Replace trusted verification keys	Key Substitution	Tampering	T1565	Verification keys are replaced with attacker-controlled keys.
	Modify stored key material	Key Tampering	Tampering	T1565	Stored cryptographic keys are altered.
	Install vulnerable firmware trust chain	Firmware Downgrade	Tampering	T1601	Older trusted keys enable vulnerable firmware installation.
Firmware Storage (D3)	Modify stored firmware packages	Firmware Tampering	Tampering	T1565	Firmware images are altered before deployment.
	Corrupt firmware repository availability	Firmware Storage Corruption	DoS	T1499	Firmware storage becomes unavailable or corrupted.
	Access stored firmware image	Unauthorized Firmware Access	Info Disclosure	T1005	Stored firmware image is accessed without authorization
	Replace legitimate firmware images	Firmware Substitution	Tampering	T1601	Malicious firmware replaces trusted firmware versions.
	Install outdated vulnerable firmware	Firmware Downgrade	Tampering	T1601	Older vulnerable firmware versions are restored.
Device Configuration (D4)	Modify device behaviour	Configuration Tampering	Tampering	T1565	Unauthorized alteration of stored configuration settings.
	Access stored device settings	Unauthorized Configuration Access	Information Disclosure	T1005	Device configuration data is accessed without authorization.
	Corrupt stored configuration data	Configuration Corruption	DoS	T1499	Configuration storage becomes unavailable or corrupted.
	Disable security protections	Security Control Bypass	Elevation of Privilege	T1078	Unauthorized modification of security-related configurations.
Insole Meta Data (D5)	Modify insole/gait data	Data Tampering	Tampering	T1565	Stored insole/gait data is accessed without authorization.
	Access stored insole/gait information	Unauthorized Data Access	Information Disclosure	T1005	Stored gait and device data is accessed without authorization.
	Delete stored insole/gait history	Data deletion	Tampering	T1565	Unauthorized removal/corruption of stored insole/gait records
	Correlate patient/device identity	Metadata Exposure	Information Disclosure	T1005	Device metadata is exposed to unauthorized parties.
App Setting Storage (D6)	Access stored application settings	Unauthorized Configuration Access	Information Disclosure	T1005	Application settings are accessed without authorization.
	Modify application configuration	Configuration Tampering	Tampering	T1565	Stored application settings are altered without authorization.
	Corrupt stored application settings	Configuration Corruption	DoS	T1499	Stored settings become unavailable or corrupted.
	Disable application security controls	Security Control Bypass	Elevation of Privilege	T1078	Security-related settings are modified improperly.
Credential & Key Storage (D7)	Access stored credentials or keys	Credential Extraction	Information Disclosure	T1552	Stored credentials or cryptographic keys are extracted.
	Modify stored authentication material	Credential Tampering	Tampering	T1565	Stored credentials or keys are altered.
	Corrupt credential storage availability	Credential Deletion	DoS	T1499	Credential storage becomes unavailable or corrupted.
	Replace trusted authentication keys	Key Substitution	Tampering	T1565	Trusted authentication keys are replaced.
	Bypass authentication protections	Security Control Bypass	Elevation of Privilege	T1078	Modified credentials bypass security restrictions.
Patient Data Storage (D8)	Access stored patient records	Unauthorized Data Access	Information Disclosure	T1005	Patient records are accessed without authorization.
	Modify patient records	Patient data Tampering	Tampering	T1565	Stored patient data is altered.
	Delete patient history	Data Deletion	Tampering	T1565	Historical patient records are deleted or corrupted.
	Bypass patient-access restrictions	Authorization Bypass	Elevation of Privilege	T1068	Access controls are bypassed to retrieve additional records.
	Correlate patient identity information	Metadata Exposure	Information Disclosure	T1005	Patient identifiers or metadata are exposed.
Credential Database (D9)	Access stored user credentials	Credential Extraction	Information Disclosure	T1552	Unauthorized extraction of stored credentials from the database.
	Modify stored authentication data	Credential Tampering	Tampering	T1565	Stored credentials are altered before authentication.
	Corrupt credential database availability	Credential Storage Corruption	DoS	T1499	Credential database becomes unavailable or corrupted.
	Reuse compromised authentication data	Credential Abuse	Spoofing	T1078	Stolen credentials are reused for unauthorized access.
	Gain access to restricted credentials	Privilege Escalation	Elevation of Privilege	T1068	Modified credentials grant elevated access rights.
Audit Log Storage (D10)	Access stored audit records	Unauthorized log access	Information Disclosure	T1005	Audit records are accessed without authorization.
	Modify stored audit records	Log Tampering	Tampering	T1565	Audit records are altered to hide malicious activity.
	Remove evidence of malicious actions	Log deletion	Repudiation	T1070	Audit records are deleted to prevent traceability.
	Corrupt audit log availability	Logging Disruption	DoS	T1499	Audit log storage becomes unavailable or corrupted.
**(d) Data Flows** **Sensor Zone (Internal Data Flows)**					
**Data Flow**	**Attacker Goal**	**Threat**	**STRIDE**	**MITRE ID**	**Description**
F2: Pairing Device Setup (P1 → P2)	Pair rogue device	Pairing Man-in-the-Middle (MITM) Attack	Spoofing	T1557	Attacker intercepts BLE pairing commands to bond a rogue device.
	Extract pairing keys	Eavesdropping	Information Disclosure	T1040	BLE pairing exchange is sniffed to recover pairing keys.
F3: Device Configuration (P2 → D4)	Access device configuration data	Configuration leakage	Information Disclosure	T1005	Device configuration data is exposed during transfer
	Modify device configuration	Configuration Tampering	Tampering	T1565	Device configuration parameters are maliciously altered before storage.
F4: Raw Sensor Data (P1 → D1)	Intercept raw gait/insole data	Data Interception	Information Disclosure	T1040	Raw sensor data is intercepted during transfer.
	Modify sensor measurements	Data Tampering	Tampering	T1565	Attacker modifies raw sensor values before storage.
F5: Pairing Configuration (D4 → P3)	Modify pairing parameters	Configuration Tampering	Tampering	T1565	Attacker alters the pairing configuration before processing.
	Access pairing configuration data	Configuration Leakage	Information Disclosure	T1005	Pairing configuration data is exposed during transfer.
F6: Buffered Sensor Data (D1 → P3)	Access buffered sensor data	Data Interception	Information Disclosure	T1005	Buffered sensor data is accessed before processing.
	Modify buffered sensor data	Data Tampering	Tampering	T1565	Buffered sensor data is intercepted and maliciously altered before processing.
F7: Load Firmware Image (D3 → P4)	Execute malicious firmware	Firmware Substitution	Tampering	T1601	Malicious firmware is loaded during boot.
F8: Store Firmware (P5 → D3)	Extract firmware image	Firmware Interception	Information Disclosure	T1040	Firmware image is intercepted during transfer, with implications for vulnerability assessment.
	Modify firmware package	Firmware Tampering	Tampering	T1565	Firmware image is maliciously modified before storage.
F9: Verification Keys (P4→D2)	Accept malicious firmware	Key substitution	Tampering	T1565	Verification keys are replaced before validation to enable malicious firmware deposit.
	Extract verification keys	Key Extraction	Information Disclosure	T1552	Verification keys are exposed during transfer.
**Sensor ↔ Mobile Boundary (BLE)**					
F10: Firmware Update (P8 → P5)	Install malicious firmware	Firmware Tampering	Tampering	T1565	Firmware image is modified during BLE transfer.
	Downgrade firmware version	Replay Attack	Spoofing	T1550	Older firmware update is replayed to the device.
	Block firmware update delivery	Update Communication Disruption	DoS	T1499	OTA firmware delivery is disrupted over BLE.
F11: Processed Sensor Data (P3 → D5)	Access insole/gait data	Data interception	Information Disclosure	T1040	Gait parameters are intercepted over BLE.
	Corrupt gait data	Data Tampering	Tampering	T1565	Gait parameters are altered before storage.
	Replay outdated insole/gait data	Replay attack	Spoofing	T1550	Previously captured gait data is resent.
F13: Pairing info (P2 → P6)	Associate wrong device with patient	Pairing data manipulation	Tampering	T1565	Pairing information altered causing incorrect device-to-patient mapping.
	Inject rogue device identity	Device spoofing	Spoofing	T1550	Attacker introduces fake device identity into pairing data.
	Replay previous pairing session	Replay attack	Spoofing	T1550	Previously valid pairing info reused to bind unauthorized device.
	Access pairing metadata	Data interception	Information Disclosure	T1005	Sensitive pairing metadata is intercepted during transfer.
	Disrupt pairing operations	Pairing Communication Disruption	DoS	T1499	Pairing information flow is disrupted during transfer.
**Edge Zone (Mobile or Home Hub)—Internal Flows**					
F12: Stored Insole Data (D5 ↔ P6)	Access stored gait data	Data Interception	Information Disclosure	T1005	Stored gait data is accessed during transfer.
	Corrupt gait analysis	Data Tampering	Tampering	T1565	Stored gait parameters are modified before processing.
	Replay outdated insole/gait data	Replay Attack	Spoofing	T1550	Previously stored insole/gait data is reused during processing.
F17: App Settings (P10 → D6)	Modify system behaviour	Configuration Tampering	Tampering	T1565	App settings are modified before storage.
	Disable application controls	Unauthorized Configuration change	Elevation of Privilege	T1078	Modified settings bypass application restrictions.
F18: Visibility Settings (P11 → D6)	Expose restricted data	Visibility Manipulation	Tampering	T1565	Visibility rules are altered before storage.
	Access hidden patient data	Unauthorized Data Access	Information Disclosure	T1005	Visibility settings expose restricted patient data improperly.
F23: Credential Access (P12 ↔ D7)	Modify stored credentials	Credential Tampering	Tampering	T1565	Stored credentials are altered to allow unauthorized access.
	Inject forged credentials	Credential Abuse	Spoofing	T1550	Forged credentials are inserted into credential storage.
	Block authentication access	Credential Deletion	DoS	T1499	Credential records are removed or corrupted to disrupt operation.
	Deny accountability	Credential Audit Inconsistency	Repudiation	T1562	Credential changes are not properly logged or traceable, preventing evidence of any malicious actions.
	Steal user credentials	Credential Extraction	Information Disclosure	T1552	Credentials are extracted during transfer to local secure storage.
F24: Pruned Old Data (D5 → P9)	Remove critical insole/gait data history.	Data Deletion	Tampering	1565	Unauthorized removal of historical insole/gait data during pruning.
	Hide key clinical insole/gait data	Pruning Rule Tampering	Tampering	T1565	Pruning logic is altered to hide clinically significant insole/gait data.
**Mobile ↔ Cloud Boundary (Internet/https)**					
F20: Data Upload (P7 → D8)	Modify uploaded clinical data	Data Tampering	Tampering	T1565	Uploaded gait data is modified during transmission.
	Replay outdated patient data	Replay attack	Spoofing	T1550	Previously captured data are resent to cloud services.
	Intercept patient data	Data interception	Information Disclosure	T1040	Patient data is intercepted during HTTPS transmission.
	Prevent clinical data delivery	Upload Disruption	DoS	T1499	The upload communication channel is blocked or degraded to compromise system operation.
F22: Visibility Request (P11 ↔ P15)	Manipulate access requests.	Request Tampering	Tampering	T1565	Patient identifiers or visibility filters are modified.
	Replay previous visibility request	Replay attack	Spoofing	T1550	Previously valid visibility request is reused by unauthorized attacker.
	Exhaust backend resources	Request flooding	DoS	T1499	Excessive visibility requests overwhelm backend services.
	Access unauthorized data	Request spoofing	Spoofing	T1550	Forged visibility request is sent to backend to gain unauthorised access.
F21: Authentication Request (P12 ↔ P13)	Capture login credentials	Credential Interception	Information Disclosure	T1552	Authentication credentials are intercepted during transmission.
	Reuse valid credentials	Replay Attack	Spoofing	T1550	Previously captured authentication tokens are replayed.
	Modify authentication request	Credential Tampering	Tampering	T1565	Authentication requests are altered before backend validation.
	Block login	Authentication Flooding	DoS	T1499	Excessive malicious requests disrupt authentication service for genuine login.
F26: Firmware Update (P14 → P8)	Install malicious firmware	Command Spoofing	Spoofing	T1550	Forged firmware update commands are sent to edge devices.
	Modify firmware parameters	Command Tampering	Tampering	T1565	OTA parameters are maliciously modified before firmware installation.
	Replay firmware update command	Replay attack	Spoofing	T1550	Previously valid OTA commands are replayed by attacker.
	Block firmware update delivery	Update Communication Disruption	DoS	T1499	OTA update command is blocked during transmission.
**Cloud Zone (Internal Flows)**					
F25: Read/Verify Credentials (P13 → D9)	Steal credentials	Credential Extraction	Information Disclosure	T1552	Credentials are accessed from the database during authentication.
	Modify credentials	Credential Tampering	Tampering	T1565	Credentials are altered to allow unauthorized authentication.
	Prevent user authentication	Credential Deletion	DoS	T1499	Credential records are removed or corrupted causing login failure.
F27: Log Data (P16 → D10)	Destroy audit trails	Log deletion	Repudiation	T1070	Audit logs removed from storage to delete evidence of any malicious actions.
	Modify stored logs	Log Tampering	Tampering	T1565	Stored logs altered to hide attacker actions.
	Access sensitive logs	Unauthorized Log Access	Information Disclosure	T1005	Audit logs are accessed revealing sensitive system activity.
F28: Web Request (P17 ↔ D8)	Bypass backend controls	Injection Attack	Tampering	T1190	Crafted API requests manipulate backend processing.
	Access unauthorized data	Authorization bypass	Elevation of Privilege	T1068	Weak request validation allows access to restricted patient data.
	Overload backend service	Request flooding	DoS	T1499	Excessive API requests from attacker disrupts backend availability.
**Cloud** ↔ **Healthcare Provider Boundary (HTTPS)**					
F29: Visibility request (P17 ↔ P20)	Access unauthorized patient data	Request Spoofing	Spoofing	T1078	Forged requests are submitted to retrieve restricted patient data.
	Modify patient query parameters	Request Tampering	Tampering	T1565	Request parameters are modified to manipulate returned patient data.
	Intercept patient records in transit	Data Interception	Information Disclosure	T1040	Patient data is intercepted during clinician-cloud communication.
F30: Visibility request (P15 ↔ P18)	Grant unauthorized visibility access	Visibility Response Spoofing	Spoofing	T1550	Forged visibility decisions are returned to clinician systems.
	Modify visibility decisions	Visibility Decision Tampering	Tampering	T1565	Visibility responses are altered before delivery.
	Replay previous visibility response	Replay Attack	Spoofing	T1550	Previously valid visibility responses are resent by attacker.
F31: Auth Request (P13 ↔ P19)	Hijack clinician session	Token Interception	Information Disclosure	T1552	Authentication tokens are intercepted during transmission.
	Reuse valid session token	Replay Attack	Spoofing	T1550	Previously captured session tokens are replayed to gain unauthorized access.
	Modify clinician session privileges	Token Tampering	Tampering	T1565	Session tokens are altered to escalate privileges.
